# INTRODUCTION

**DOI:** 10.1002/pmf2.70192

**Published:** 2026-02-09

## Introduction to the Conference Abstracts

The Society for Maternal‐Fetal Medicine (SMFM) is dedicated to the advancement of optimal and equitable perinatal outcomes for all people who desire or experience pregnancy. Founded in 1977, SMFM is the medical professional society for obstetricians who have additional training in high‐risk, complicated pregnancies. SMFM represents more than 6,500 members who care for high‐risk pregnant people and provides education, promotes research, and engages in advocacy to reduce disparities and optimize the health of high‐risk pregnant people and their families. SMFM and its members are dedicated to optimizing maternal and fetal outcomes and assuring medically appropriate treatment options are available to all patients.

SMFM hosts the annual Pregnancy Meeting™, the premier event dedicated to advancing research and education in maternal‐fetal medicine. All abstracts submitted for the conference undergo a rigorous review by the SMFM Abstract Review Committee, with selected submissions chosen for presentation based on their relevance to maternal‐fetal medicine and obstetric care. Many of these abstracts are also eligible for SMFM's abstract awards.

Please note that the abstracts in this supplement have not been copyedited, and any errors are the responsibility of the authors. SMFM assumes no liability for inaccuracies. This content is for informational purposes only and does not constitute medical or professional advice.

For more information, visit www.smfm.org/pregnancy‐meeting.

